# Monoclonal Antibodies and Flaviviruses: a Possible Option?

**DOI:** 10.1128/mbio.00824-22

**Published:** 2022-05-16

**Authors:** Nicasio Mancini

**Affiliations:** a Laboratorio di Microbiologia e Virologia, Università Vita-Salute San Raffaele, IRCCS Ospedale San Raffaele, Milan, Italy

**Keywords:** flaviviruses, monoclonal antibodies, yellow fever virus

## Abstract

M. P. Doyle, J. R. Genualdi, A. L. Bailey, N. Kose, et al. (mBio 13:e00512-22, 2022, https://doi.org/10.1128/mBio.00512-22), report on the cloning of a panel of fully human monoclonal antibodies (mAbs) directed against yellow fever virus (YFV). In particular, mAb YFV-136 is endowed with interesting cross-YFV substrain-neutralizing features. The importance of YFV-136 and other mAbs with similar characteristics is related not necessarily only to their possible future use in the clinic but also to their role in a better understanding of the biology of YFV (as well as of other flaviviruses) for the development of effective therapeutic and prophylactic strategies. The emergence and reemergence of different flaviviruses worldwide in the last decades certainly make this a compelling clinical priority.

## COMMENTARY

Yellow fever has been for a long time, and ominously is somehow returning to be, one of the most impacting infectious diseases worldwide, with an estimated 51,000 deaths only in 2018 (1). Although its burden was dramatically limited in the last century by one of the most effective vaccines available to date (based on different “substrain” variants of the live-attenuated 17D strain developed in the 1930s), it is still a major matter of concern. This is due to several factors, including, but not limited to, the logistic difficulties related to vaccine production and the increasing reported outbreaks in areas of endemicity caused by the increasingly closer borders between the so-called “sylvan cycle” and “urban cycle” of yellow fever virus (YFV). YFV is therefore dangerously approaching densely populated areas in Africa and the South American continent. Actually, the lack of approved antiviral agents directed against YFV is still a major problem in the management of yellow fever, which is limited to supportive therapy only (2).

Under this perspective, it is therefore very encouraging what is reported by Doyle et al. (3) about the development of a cross-strain-reacting fully human monoclonal antibody (mAb) (YFV-136) directed against the YFV envelope (E) protein, that is, the viral structure mediating both viral attachment and subsequent membrane fusion. YFV-136 is endowed with potent neutralizing activity (50% inhibitory concentration [IC_50_] of <10 ng/mL) in both pre- and postattachment *in vitro* neutralization assays, showing its interference with the E-mediated membrane fusion step of the viral entry phase. This activity was evident against both the 17D vaccine strain (unfortunately, it is not clear how many and which of the three substrains currently available were used in the study) and wild-type viral strains (Asibi and Kouma). Moreover, it also featured protective activity in two different animal models (Syrian golden hamsters and immunocompromised mice engrafted with human hepatocytes) using both animal-adapted and human wild-type YFV strains. Importantly, the attention of those authors was correctly focused on the therapeutic potential of mAb YFV-136, as shown by its postinfection administration (3 days postinfection [p.i.] in hamsters and 8 h p.i. in transgenic mice) in both models, thus better recapitulating its most probable future clinical use in the case of human infections.

Other similar steps were already made in this direction with several other mAbs, including fully human ones, described in the literature. In particular, one mAb, named TY014, was already tested in a phase Ia/Ib trial (4). This mAb showed a very good safety profile and was effective in dramatically limiting the entity and the duration of viremia in healthy human volunteers inoculated with the live-attenuated YF17D-204 vaccine substrain. Although being the only study based on the administration of anti-YFV mAbs, this clinical trial had a major limitation related to the use of TY014 “only” 24 h after viral challenge. This situation is far from the possible clinical use of an anti-YFV mAb, especially due to the average of 3 to 6 days of incubation of the infection before any clinical manifestation, which may also mean a longer time before requiring any medical aid due to the low specificity of the first clinical manifestations. This point certainly deserves particular attention in the future development of YFV-136 and antibodies with similar characteristics.

Another crucial point is the correct *in vivo* dosing of the mAb, especially considering the well-known possible “side effect” of the antibody response elicited by YFV, that is, antibody-mediated enhancement of infection (5). This is possible after a second contact with YFV (as well as with other flaviviruses), especially in the case of nonneutralizing antibodies but also in the case of low titers of neutralizing antibodies. Very honestly, Doyle et al. (3) report that at “lower antibody concentrations,” a “modest enhancement” of *in vitro* infectivity was observed. However, it would certainly have been interesting to know precisely how low that “lower concentration” was. Moreover, it would certainly have been important to know how much those “lower concentrations” enhancing infectivity were somehow influenced by the infecting viral load. Obviously, the points raised above are other crucial points that the authors will certainly bear in mind in the future development of their mAb.

Not only is the definition of the epitope bound by cross-neutralizing mAbs certainly important from a speculative point of view, but it also can pave the way to the possible development of more tailored vaccinal approaches (6). The preliminary definition of the epitope recognized by YFV-136 and its core identified within E protein domain II (DII), in proximity to the fusion loop, is fully coherent with the features observed in the postattachment settings of the neutralization assays. A finer definition using both wet and *in silico* analyses is certainly needed to shed more light on both the structural and kinetic features of the mAb-antigen interaction. However, there is certainly another point emerging from the paper, which deserves a dedicated comment. A single round of infection *in vitro* was sufficient to select for an escape viral mutant capable of evading neutralization by YFV-136, even at a 1,000-fold-higher concentration than the previously calculated IC_50_. The identification of a single histidine-to-tyrosine substitution at position 67 within DII further confirms that this domain is important for YFV-136 binding and neutralizing activity. At the same time, this shows how easily this residue can be mutated without apparently affecting viral infectivity (specific data are not reported, but a possible decrease in viral fitness would certainly have been highlighted by the authors). The consequence of this observation is of pivotal importance from a clinical point of view, dramatically limiting the possible future use of YFV-136, as well as other similar mAbs, in monotherapy. The risk of the selection of viral escape variants has to be considered, especially in the case of RNA viruses such as flaviviruses, and its limitation through tailored strategies (i.e., the combined use of neutralizing mAbs targeting different epitopes) should be something somehow considered already early in preclinical development.

Finally, the interesting paper by Doyle et al. (3) also gives the opportunity to speculate on a more general level about the possibility of selecting mAbs (as well as other types of molecules with possible neutralizing activity) that not only are cross-reactive against different YFV strains but also have “cross-flavivirus”-neutralizing potential (7). This is extremely intriguing, especially considering that “novel” flaviviruses have been emerging in the last decades (i.e., Zika virus [ZIKV], Japanese encephalitis virus [JEV], tick-borne encephalitis virus [TBEV], and Usutu virus [USUV]) and that even more are expected to make their appearance in the future (8). However, quasi-atomic-resolution viral particle structures of several flaviviruses evidenced high heterogeneity not only among different flaviviruses but also among viral particles resulting from a single infection (9). This is probably due to the differential proteolytic maturation of the E protein and its high plasticity (“viral breathing”) once on the viral surface. These movements may lead to the exposure of different epitopes during a single infection, including “cryptic epitopes” not easily accessible to the immune response and not easily studied using static structural approaches. Some of these epitopes may somehow be shared among different flaviviruses (10). The task of looking for safe (i.e., not inducing viral enhancement) cross-flavivirus mAbs is therefore very demanding, but theoretically not impossible, and should necessarily consider the point mentioned above.

The continuously reemerging threat of YFV, as well as of other flaviviruses, forces the development of effective therapeutic strategies to be used during outbreaks. To reach this goal, a deeper comprehension of the biological details of flavivirus replication is certainly needed, together with a better understanding of the immune response elicited. The development of the mAbs described by Doyle et al. (3) absolutely goes in this direction, and this and other similar studies may further contribute to the identification of protective epitopes possibly targeted by other non-mAb-based therapeutic approaches and useful in the development of novel prophylactic strategies.
